# Reliable Measurements of the **β**-Amyloid Pool in Blood Could Help in the Early Diagnosis of AD

**DOI:** 10.1155/2012/604141

**Published:** 2012-08-16

**Authors:** Pedro Pesini, Virginia Pérez-Grijalba, Inmaculada Monleón, Mercè Boada, Lluís Tárraga, Pablo Martínez-Lage, Itziar San-José, Manuel Sarasa

**Affiliations:** ^1^Araclon Biotech Ltd., I + D Laboratory, Zaragoza, Spain; ^2^Araclon Biotech Ltd., Proteomic Laboratory, CIBIR Logroño, Spain; ^3^Alzheimer Research Center and Memory Clinic, Fundació ACE, Institut Català de Neurciències Aplicades, Barcelona, Spain

## Abstract

The present study was aimed at assessing the capability of A**β**1-40 and A**β**1-42 levels in undiluted plasma (UP), diluted plasma (DP), and cell bound (CB) to distinguish between early stages of Alzheimer's disease (AD), amnesic mild cognitive impairment (MCI), and healthy control (HC). Four blood samples from each participant were collected during one month and the levels of A**β**1-40 and A**β**1-42 were determined by a blinded proprietary ELISA sandwich (Araclon Biotech. Zaragoza, Spain). First striking result was that the amount of A**β**1-40 and A**β**1-42 in UP represented only a small proportion (~15%) of the total beta-amyloid pool in blood (**β**APB) described here as the sum of A**β**1-40 and A**β**1-42 in blood where they are free in plasma, bound to plasma proteins, and bound to blood cells. Furthermore, we found that levels of A**β**1-40 and A**β**1-42 in UP, DP, and CB were significantly higher in MCI when compared to HC. On average, the total **β**APB was 1.8 times higher in MCI than in HC (*P* = 0.03) and allowed to discriminate between MCI and HC with a sensitivity and specificity over 80%. Thus, quantification of several markers of the **β**APB could be useful and reliable in the discrimination between MCI and HC.

## 1. Introduction

Histopathological hallmarks of AD are senile plaques and neurofibrillary tangles. It is becoming increasingly clear that development of these lesions appears to take place over a period of many years before any measurable cognitive impairment can be detected [1]. Thus, there is an urgent need for biomarker-based tests that allow a more accurate and early diagnosis of the disease. Such tests should improve the monitoring of the disease progression, enable early therapeutic intervention (before irreversible neurodegeneration has taken place), allow a better selection of AD cohorts for clinical trials, and serve for the evaluation of new AD therapies, particularly amyloid targeting drugs and vaccines [2].

Recently it has been shown that CSF levels of A*β*1-42, total tau, and phosphorylated-tau proteins define a biomarker signature that is diagnostic for AD and appears to predict conversion from MCI to AD [2–6]. Indirectly, these results reinforce the idea that A*β* blood levels could also have diagnostic significance, although the relationship among brain, CSF, and plasma peptide levels in health and disease is complex and far from being thoroughly understood [7–9].

The practical advantages of a blood test over a CSF test are clear and do not need to be stressed. However, until now attempts to measure A*β* peptides in blood have produced contradictory and discouraging results, mostly for a variety of technical reasons that certainly compromise but, in our opinion, do not invalidate the working hypothesis [10–12]. Indeed, it is well established that A*β* blood levels are elevated before the onset of dementia in dominant-inherited early AD and Downs syndrome [13, 14]. Levels of A*β* peptides have also been found to be elevated in healthy elderly people before late-onset AD and in people with high risk of developing AD, such as women with MCI and first-degree relatives of patients with late-onset AD [15–17]. Furthermore, it has been recently shown that higher plasma levels of A*β*1-42 in healthy people are associated with increased risk of developing AD in the follow-up period [16, 18, 19]. Interestingly, several studies suggest that conversion to AD is followed by a significant decline in plasma A*β* peptide levels after the onset of dementia and with progression of symptoms [15, 16, 19–21]. The consensus arising from these papers is congruent with studies in an AD mouse model (tg 2576), which have shown highly significant decreases in plasma A*β*1-40 and A*β*1-42 linked to the marked accumulation of A*β* in the brain that begins in these animals at 6–9 months of age [22].

Regarding A*β* blood tests, it is also important to take into account that due to the biochemical nature of A*β* peptides, particularly their extreme hydrophobicity, they will be found free in the plasma, bound to plasma proteins, and bound to blood cells [23–25]. Thus, any A*β* blood test should consider the amount of peptides in each of these three fractions, which we have described collectively as the *β*-amyloid pool in blood (*β*APB); a concept that certainly will evolve because it is known that apart from A*β*1-40 and A*β*1-42, other A*β* species are present in blood and experimental results suggest that they could play a role in the disease and may also be of value in the diagnosis [26].

The present work is aimed at exploring whether measurements of the different fractions of the *β*APB could provide reliable and useful biomarkers for MCI and early AD stages.

## 2. Material and Methods

### 2.1. Study Population

The study included 40 participants: 16 healthy controls (HC), 8 amnesic mild cognitive impairment patients (MCI), and 16 Alzheimer's disease patients (AD). All were over 65 years of age and each group comprised 50% male and 50% female. Demographic characteristics of the participants are summarized in Table 1.

Healthy controls were carefully selected from community-dwelling, socially active volunteers (no blood-relatives to the patients) with an absence of memory complaints, normal performance in neuropsychological tests, and absence of structural alterations in quantitative magnetic resonance imaging (MRI).

Participants with MCI fulfilled the Mayo Clinic criteria. Additional requirements for the selection of MCI participants included a clinical dementia ratio (CDR) of 0.5 points, more than 3 points on the Scheltens scale for medial temporal atrophy (MTA) [27] and a pattern of parietal and/or temporal hypometabolism in positron emission tomography with 18-fluorodeoxyglucose (PET-FDG) suggestive of AD. Patients with any psychiatric or systemic pathology, other than possible neurodegenerative disease, that could cause the MCI were excluded. Specific inclusion criteria for the AD group were a diagnosis of probable AD (NINCDS-ADRDA criteria), a CDR of 1 point, a mini-mental state examination (MMSE) between 16 and 24 points, and more than 3 points on the Scheltens scale for medial temporal atrophy.

Cognitive testing for HC, MCI, and AD diagnosis was performed according to the routines of the Memory Clinic of Fundació ACE as described elsewhere [28].

Written informed consent was obtained from every participant or their closest relative in the case of several AD patients. The study protocols were reviewed and approved by the Ethical Committee of the Hospital Clinic i Provincial (Barcelona, Spain).

### 2.2. Blood Sampling and Biochemical Determinations

Four blood samples (BS1 to BS4) per participant were obtained in the morning after an overnight fast for four consecutive weeks. Blood samples were collected in polypropylene vials with EDTA and protease inhibitor cocktail (Complete Mini, Roche); they were immediately centrifuged, aliquoted, and coded without any reference to the cognitive state of the participant. Two independent 5 mL blood samples from each participant were collected for ApoE genotyping and clinical blood analysis including cell-hemogram. The aliquots of plasma and the remaining cell pellet were immediately frozen at −80°C and sent to the Araclon laboratory. APOE genotyping was performed by amplification of genomic DNA, digestion with HhaI, and analysis of the restriction fragments [29]. A*β*1-40 and A*β*1-42 were measured in plasma and cell fractions using two specific ELISA sandwich kits, ABtest 40 and ABtest 42 (Araclon Biotech, Zaragoza, Spain). Before analysis, plasma and blood cell samples were pretreated by 1 : 3 dilution in a formulated sample buffer (PBS 0.5 M, 0.5% Tween-20, 1% blocking polymer) according to the supplier's instructions. An anti-A*β* N-terminal monoclonal antibodies was used as capture antibody and two highly specific anti C-terminal polyclonal antibody, pAB002 and pAB031 (Araclon Biotech, Zaragoza, Spain), were used as detection antibody for A*β*1-40 and A*β*1-42, respectively. Thus, levels of A*β*1-40 and A*β*1-42 were separately determined in undiluted plasma (UP), diluted plasma (DP) and cell bound (CB) fractions. Briefly, samples were incubated overnight with capture antibody at 4°–8°C without shaking. Subsequent incubations (1 hour each) were performed at room temperature gently shaking the plates in an orbital 3D platform and were followed by 5 minutes washing. During last incubation with the chromogen, the absorbance was read at 620 nm until it reached 0.60–0.65. Then, the reaction is stopped and the absorbance read at 450 nm for interpolation in the standard curve. Subsequently, aliquots of sixteen randomly chosen samples from any extraction and participant were sent to two clinical diagnostic laboratories for the evaluation of inter-laboratory reproducibility of the measurements. Assays were independently performed following the ABtest 40 and ABtest 42 data sheets by separate personnel and equipment. Samples and peptide standards were always assayed in triplicate.

### 2.3. Statistic Analysis

**Figure 1:**
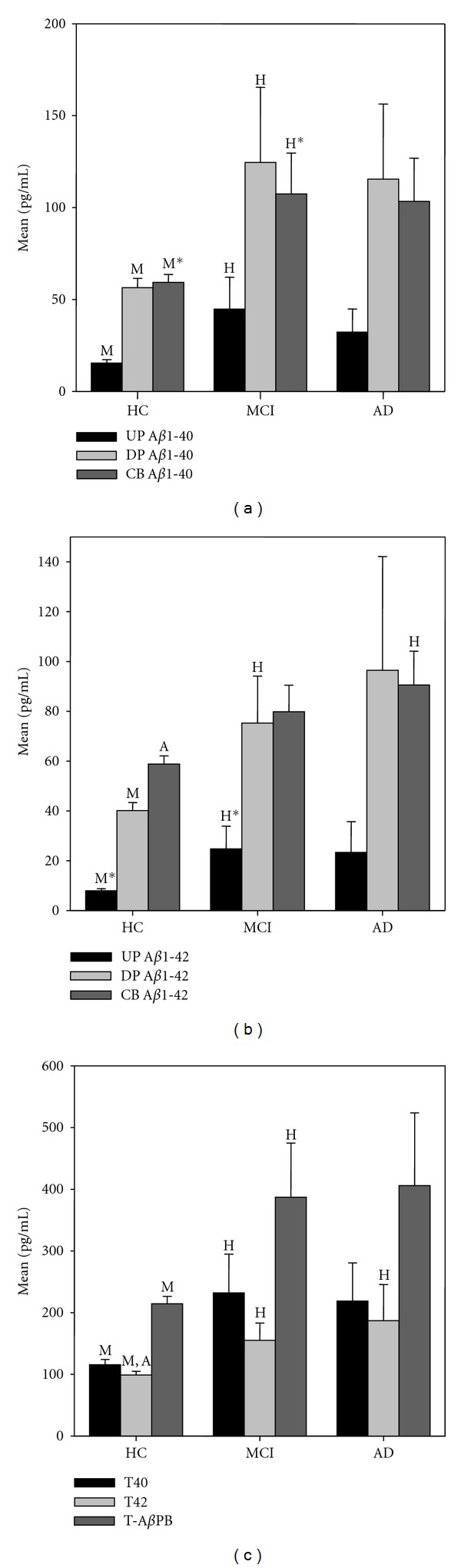
((a)–(c)). Bar graphs of (a) A*β*1–40, (b) A*β*1-42 direct markers and (c) T40, T42, and total *β*APB (T-A*β*PB) calculated markers. H, M, and A represent significance (*P* < 0.05) with regard to HC, MCI, and AD, respectively. *means *P* < 0.01.

**Figure 2:**
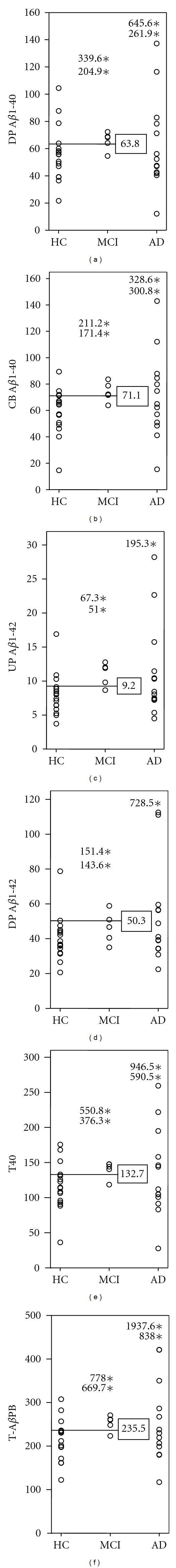
((a)–(f)). Dot-plot for (a) DP A*β*1-40, (b) CB A*β*1-40, (c) UP A*β*1-42, (d) DP A*β*1-42, (e) T40 and (f) T-*β*APB values in HC, MCI and AD participants. Numbers beside *indicate the value of outliers in the MCI and AD groups, which, for clarity of representation, are not represented at the same scale of the ordinate axis. The horizontal line with the number in parentheses represents the cut-off value between MCI and HC.

Interlaboratory reproducibility of the measurements was assessed by the concordance correlation coefficient (CCC) which evaluates the agreement among the three readings from the same sample, our own, and those reported by the two external laboratories, measuring the deviation of the reading from the 45° line through the origin (the concordance line). This is written Rc = *R* × Cb. The term *R* is the standard Pearson correlation coefficient, while Cb (bias correction factor) = 2/(*v* + 1/*v* + *u*²) where *v* = *s*1/*s*2 and *u* = (*m*12212*m*2)/SQRT(*s*1 × *s*2; *m*1, *m*2 and *s*1, *s*2 being the mean and standard deviation of the first and second set of measurements in each comparison [30]. Intrasubject reproducibility along the four blood samples was assessed by the intra-class correlation coefficient (ICC). The degree of agreement estimated by these correlation coefficients was described as poor (0.21 to 0.40), moderate (0.41 to 0.60), substantial (0.61 to 0.80), or almost perfect (0.81 to 1.00). The A*β* levels in the different diagnostic groups were compared using the Mann-Whitney *U* test. The categorical variables (education level and ApoE4 genotype) were compared by Chi-square test. Spearmans analysis was used to evaluate correlations among continuous variables. *P* < 0.05 was required for rejection of the null hypothesis. All statistical analysis, including receiver operating characteristic (ROC) curve analysis, was performed with SAS 9.1 software. Graphs in Figures 1–2 were generated with SigmaPlot 11.0 software. Lack of normality, linearity and homoscedasticity in the distribution of the A*β* marker values prevented the statistical comparisons between groups to be reliably adjusted for the age of the participants.

## 3. Results

### 3.1. Inter- and Intralaboratory Reproducibility of A*β* Measurements

Sixteen randomly chosen samples from any participant and blood sampling were sent to two external laboratories for the evaluation of interlaboratory reproducibility of the measurements. The six markers directly assayed in the samples (A*β*1-40 and A*β*1-42 for UP, DP, and CB) behaved in a similar way with a CCC that ranged from 0.84 to 0.99 (overall 95% CI from 0.73 to 0.99) which corresponds to a degree of agreement between substantial to almost perfect in all cases (complementary material, Table 2).

Average intra-assay reproducibility, expressed as the coefficient of variation of the triplicate wells, for the six markers in each laboratory was 4.31, 5.83, and 8.34 (complementary material, Table 3). The lower limit of detection (LLD) of the assays in the three laboratories was 5.31, 3.63, and 1.91 pg/mL for A*β*1-40 and 2.37, 2.04, and 2.45 pg/mL for A*β*1-42.

### 3.2. Intra-Individual Reproducibility of A*β* Measurements

The reproducibility of A*β* measurements among the four weekly blood collections (BS1–BS4) as measured by the ICC varied between substantial to almost perfect for all the direct markers in the three groups (complementary material, Table 4). Generally, for the three diagnostic groups, the higher ICC corresponded to the measurements of A*β*1-40 and A*β*1-42 in DP; these were 0.93 (95% CI = 0.98–0.80) and 0.93 (95% CI = 0.98–0.78), respectively.

### 3.3. Comparison between Diagnostic Groups

In concordance with the high intrasubject reproducibility of the measurements, comparison between groups of participants followed the same pattern in the four blood samples collected on different days (BS1 to BS4), although some *P* values varied slightly from one BS to another. Check Table 2 is based on the measurements of BS4.

The first striking result was that the concentration of UP A*β*1-40 and UP A*β*1-42 represented only approximately 1/3 and 1/4 of the levels in DP for any diagnostic group, respectively (Table 2). Second, the CB peptide levels, which were directly measured from the cellular fraction of the blood sample, were similar to the levels measured in the DP. Moreover, levels of A*β*1-40 and A*β*1-42 strongly correlated when measured in either UP, DP or CB (*r* = 0.58, 0.71 and 0.71, resp *P* < 0.001). Significant correlations were also found between any pair of the six markers directly assayed in the samples (A*β*1-40 and A*β*1-42 in UP, PD, CB; Table 3).

Furthermore, we found that levels of every marker increased in MCI and AD patients compared to the healthy control group (Figure 1, Table 2). These increases reached statistical significance between the MCI and HC groups for the three A*β*1-40 markers (UP, DP, and CB which increased 2.9, 2.2 and 1.8 times resp.) and for the two A*β*1-42 plasma markers (UP and DP which increased 3.1 and 1.8 times resp.). The average level of every marker in the AD group was very similar to its average level in the MCI group and no significant differences were found between these two groups of patients. 

Both A*β*1-40 and A*β*1-42 plasma markers (UP and DP), but not CB, correlated significantly with MMSE (Table 3), although it could be overestimated in part because of the clustering of HC toward the higher MMSE scores. In fact, when participants with MMSE ≥ 26 were excluded, levels of A*β*1-40 and A*β*1-42 were lower in severely affected patients (MMSE ≤ 21, *n* = 5) than in moderately affected patients (MMSE 22–25, *n* = 12), although the differences did not reach statistical significance (data not shown). Additionally, the levels of A*β*1-42 in UP and DP were found to significantly correlate with the degree of MTA in both the right and the left hemispheres (Table 3). 

We also considered several markers that were calculated from those assayed directly from the samples. Apart from the usual A*β*1-42/A*β*1-40 ratios, the most interesting were the sum of DP plus CB A*β*1-40 and the sum of DP plus CB A*β*1-42, which we defined as total A*β*1-40 (T40) and total A*β*1-42 (T42), respectively; and the sum of these two, which we labeled total *β*-amyloid pool in blood (T-*β*APB). The A*β*1-42/A*β*1-40 ratios, measured in UP, DP or CB, did not show significant differences between groups. However, the T40, T42 and T-*β*APB increased 2.0, 1.5 and 1.8 times, respectively in the MCI group compared to the healthy control group (*P* ≤ 0.03) (Table 2). Similar average increases were found between HC and AD patients but in this case only T42 reach statistical significance (Figure 1). 

### 3.4. Sensitivity and Specificity of the Direct and Calculated Markers

ROC curve analysis was used to assess the sensitivity and specificity of each marker (Table 4). Most direct markers and two calculated markers (T40 and T-*β*APB) met the criteria considered suitable to distinguish between MCI patients and HC. Thus, all the A*β*1-40 markers, and UP A*β*1-42, presented an area under the ROC curve ≥ 0.80. The calculated T40 and T-*β*APB were equally as accurate as the direct markers in distinguishing between MCI and HC, whereas T42 reached lower specificity (Table 4). Due to the great variability of A*β* measurements from one individual to another within the AD group, no critical point could be found at which these markers differentiated the AD patients from the other two groups of participants with an acceptable sensitivity and specificity (Figure 2). 

## 4. Discussion 

Quantifying A*β* peptide levels in blood by ELISA is no doubt a demanding laboratory task; it requires considerable expertise due to a variety of technical reasons that compromise obtaining reliable and reproducible results. Demanding conditions of the assays and complexity of the procedures are mostly related to the relative low levels of these peptides in the blood and their high hydrophobic nature [11, 31, 32]. Due to the low levels of A*β* peptides in blood, it is critical to use antibodies with high affinity and to strictly standardize the methodology for the A*β* blood test, as has been done for CSF test in the framework of the Alzheimer Disease Neuroimaging Initiative (ADNI) (http://www.adni-info.org/) and other projects (http://www.neurochem.gu.se/theAlzAssQCProgram) from sample collection to final assay [33]. Our results show that if sufficient attention is devoted to these issues, the ELISA sandwich colorimetric test is sensitive enough to detect low concentrations of A*β* (LLD < 6 pg/mL) with very good intra-assay repeatability and interlaboratory reproducibility, even with a relatively low number of samples. 

The biochemical properties of A*β* peptides make them very difficult molecules to work with due to their tendency to self-aggregate and to develop hydrophobic interactions with any other molecules in their vicinity. In the present work, we systematically analyzed, for the first time, the levels of A*β* peptides in UP, DP, and CB. We hypothesized that the levels of A*β* peptides measured in UP could correspond mainly to the amount of peptides free in the plasma. Dilution of the plasma with sample buffer changed the ionic strength and molecular interactions within the sample, leading to the release of A*β*1-40 and A*β*1-42 bound to plasma protein and other components [31, 34]. Thus, the increase in the measurements after dilution of the plasma might be due to the detection of A*β* peptides released from proteins and other plasma components and could be interpreted as an estimation of the total level of A*β* in plasma [6, 23, 25]. Theoretically, the possibility that the breakage of A*β* oligomers present in plasma might contribute to this increase could not be excluded and will be the object of future research [19, 20]. Further, we found that around 50% of the total *β*APB was bound to the cellular pellet remaining after centrifugation, a fraction usually not considered for A*β* quantification. This finding is consistent with previous reports of A*β* peptides bound to erythrocytes by complement receptor C1, which mediates a significant proportion of A*β* clearance from the bloodstream [24]. In any case, these results show that A*β* peptide levels in blood could be much higher than the levels usually reflected by most ELISAs which do not take into account the substantial amounts of peptides bound to plasma proteins and blood cells. From a practical point of view, this might not be a major problem since in the present study, levels of A*β*1-42 free in the plasma reached by themselves a sensitivity and specificity enough to distinguish between MCI and HC. However, it should be considered that UP A*β*1-42 levels, particularly in healthy controls are very low, close to the LLD of most ELISA kits, which many times impairs the reliability of these measurements. Our results suggest that other more substantial and reliable markers as DP and CB A*β*1-40 and even DP A*β*1-42 could enable also a suitable differentiation between HC and MCI patients. Thus, a complete determination of the total *β*APB blood levels should include the quantification of the peptides that are free in plasma, bound to plasma proteins and bound to blood cells. This comprehensive quantification of the different components of the *β*APB would give a more precise measurement of A*β* blood levels and might help to ascertain the complex regulation of A*β* peptides in health and disease. 

Mounting evidence is reinforcing the idea that A*β* blood levels are affected in MCI and AD patients, although controversy remains based on some contradictory reports. In the present work, samples from MCI or AD participants, when compared to HC, showed a considerable increase in the detection of six directly assayed *β*APB markers. For five of these six markers, the increases were statistically significant between HC and MCI, but not between AD and HC. Nevertheless, caution should be exerted interpreting these results since comparisons were not adjusted for age. It should be taken into account that adjustment for age might be by itself a confounding factor in transversal studies fading possible differences between healthy controls and MCI patients. Age is the main risk factor for AD and experimental results are increasingly supporting that beta-amyloid modifications, detected either by PET-PIB or CSF ELISA assay, are the earliest sings of AD amyloid pathology which could be in place many years before the first cognitive symptom could be detected. Furthermore, it is believed that A*β* levels reach a kind of plateau and do not keep increasing as disease progress [35]. Thus in transversal studies, increasing the age of the participant (or statistically adjusting the weight of the younger controls) would increase the possibility of misplacing patients with cognitively-asymptomatic-AD amyloid pathology within the “healthy control” groups (or increasing their statistical weight), which might fade the presumptive differences in A*β* markers between “healthy control” and MCI patients. 

The present results are consistent with numerous studies on late-onset AD that reported a relationship between elevated plasma A*β* peptide levels and disease [15, 16, 18–21, 32, 36–38]. It is becoming clear from these studies that increases in A*β* blood levels is an early event that precedes the onset of cognitive symptoms and increases the risk of developing AD. Studies with MRI and PET-PIB also support the idea that an individual's amyloid load accumulates prior to clinical symptoms and reaches a plateau with no further or very slow accumulation as the disease progresses clinically [39]. This idea is also supported by experimental results from early-onset familial AD and Downs syndrome patients who develop dementia of the Alzheimer type and from studies in transgenic mouse models of the disease [14, 17]. Interestingly, several studies report significant decreases in A*β* blood levels (again more marked for A*β*1-42 than for A*β*1-40) as the disease progresses [15, 16, 19–21]. Our results also suggest that when MCI can be clinically detected, the different markers of the *β*APB tend to stabilize at levels higher than normal on average and then tend to decrease in patients with lower MMSE. 

Lack of significance between our AD and HC groups is most likely due to the large range of measurements in the AD group, in which levels of the different markers varied considerably from one individual to another (2.5 to 5.7 times the coefficient of variation observed in the HC). In fact, our results showed a clear increase in the coefficient of variation of the different markers as the disease progresses from HC to MCI and AD groups (Table 2). This variability of A*β* blood levels within the AD group might reflect an actual characteristic of the disease and could be related to individual features like the presence of other systemic or neurological pathologies, including the affectation of cerebral blood vessels and blood brain barrier, which frequently accompany AD [21, 40]. Reports on increases in A*β* blood levels in early stages of AD have been sometimes interpreted as contradictory to the well defined CSF biomarker signature of AD, which demonstrates a significant decrease in A*β*1-42 [4, 6]. In our opinion, this discrepancy could have been misinterpreted and could be likely related to a different temporal sequence of changes in A*β* peptide levels in blood compared to CSF. Thus, A*β* produced by cortical neurons has to travel a long distance through the interstitial fluid (ISF) to reach the ventricular system located deep within the brain, at least several millimeters away. During this travel along facilitated routes passing by fiber tracts and blood vessels, A*β* will have to cross a huge number of capillaries that thoroughly pierce brain tissue ~40 *μ*m from each other [41]. The amyloid hypothesis states that at the onset of AD pathology, A*β* peptides start to aggregate and precipitate in the interstitial space of the brain. In this situation, diffusion of A*β* in the ISF toward the ventricular system will be severely hampered, leading to a reduction of A*β*1-42 levels in CSF that herald cognitive decline. At this initial stage, without extensive capillary damage, the increased A*β* blood levels observed in the present work and previous reports might reflect the increased peptide levels in brain tissue. Nevertheless, as suggested by reports from longitudinal studies, A*β* blood levels would finally drop in most patients probably after the deposition of A*β* around capillaries would seal off the BBB. The fact that in the present pilot study, the levels of A*β*1-40 and A*β*1-42 in the most severely affected patients (MMSE ≤ 21, *n* = 5) were lower than those in moderately affected patients (MMSE 22–25, *n* = 12) is congruent with this interpretation. Further experiments are needed to understand the circulation of A*β* between ISF, CSF, and blood as well as the relationship between biochemical and neuroimaging biomarkers [35, 42–46]. 

Our results do not support previous studies that reported a lack of a relationship between A*β* plasma levels and risk of AD or an association between either low plasma A*β* levels or low A*β*1-42/A*β*1-40 ratio and AD [6, 47–51]. Interestingly, some of these studies were aimed at comparing healthy controls to overt AD patients or pooled MCI and AD patients within the same group [47–49]. These inconsistencies could most likely be accounted for by differences in the timing of sample collection with regard to the onset of the disease and the stage of progression. 

It is still too early to clearly establish the role of the A*β* blood test as a diagnostic tool for MCI and AD. Nevertheless, in the present work we have shown that most direct markers of the *β*APB and two calculated markers (T40 and the total *β*APB) reported promising results to distinguish between MCI patients and HC. This could be of utmost interest because from any practical point of view it is there were the diagnostic should be improved. However, the small sample size of this pilot study did not allow definitive conclusion on this regard but most markers of the *β*APB showed consistent results that will be useful to adequately estimate the sample size for further statistically well-powered analysis of the diagnostic capabilities of these biomarkers. 

In summary, the A*β* blood test should include the quantification of the peptides that are free in plasma, bound to plasma proteins and bound to blood cells. Provided there is a well-established standardization of the procedures, the ELISA test can produce reliable measurements with high repeatability, low limits of detection, and substantial inter-laboratory reproducibility. Under these conditions, the levels of several direct and calculated markers of the *β*APB appear to be useful for the diagnosis of mild cognitive impairment and their quantification should be included in the ordinary analytical battery of tests on aged patients with memory complaints. 

## Figures and Tables

**Table 1:** Demographic characteristics.

Characteristic	HC	MCI	AD	*P*
Male/female	8/8	4/4	8/8	—
Age (years, mean ± SD)	70.3 ± 4.1^M,A^	77.3 ± 3.6^H^	78.8 ± 4.7^H^	0.0002^∗^
ApoE *ε*4 carrier	6%	62%	62%	0.002^§^
Education level (undergraduate/ graduate)	50%/50%	87%/13%	62%/38%	0.202^§^

^∗^ Kruskal-Wallis *U* test, contrasts with Mann-Whitney *U* test. H, M, and A mean significant with regard to HC, MCI, and AD, respectively. ^§^Chi-square test.

**Table 2:** Levels of direct and calculated markers in each group of participants.

Marker	HC				MCI				AD			
Direct	*n*	Mean	CV	Range	*n*	Mean	CV	Range	*n*	Mean	CV	Range
UP A*β*1-40	16	15.4^M^	47.4	2.2–33.3	7	44.7^H^	102.7	14.4–133.6	15	32.2	152.5	7.2–203.2
DP A*β*1-40	16	56.4^M^	36.2	21.4–104.3	7	124.6^H^	86.9	54.5–339.6	15	115.4	137.3	12.0–645.7
CB A*β*1-40	16	59.3^M*^	28.8	14.5–89.2	7	107.4^H*^	54.7	63.7–211.2	15	103.3	88.2	15.3–328.6
UP A*β*1-42	16	8.0^M*^	38.7	3.7–16.9	7	24.8^H*^	96.8	8.6–67.4	15	23.3	206.0	4.5–195.3
DP A*β*1-42	16	40.1^M^	32.2	20.5–78.6	7	75.2^H^	66.5	34.9–151.5	15	96.5	183.3	22.3–728.5
CB A*β*1-42	16	58.7^A^	23.2	28.4–76.8	7	79.8	35.5	62.9–141.9	15	89.8^H^	59.0	52.6–262.6

Calculated												

UP A*β*-42/A*β*40	16	0.7	85.7	0.1–2.9	7	0.6	16.7	0.5–0.7	15	0.6	50.0	0.3–1.2
DP A*β*-42/A*β*40	16	0.8	25.0	0.3–1.5	7	0.7	14.3	0.4–0.9	15	0.9	55.5	0.4–2.6
CB A*β*-42/A*β*40	16	1.1	63.6	0.4–3.7	7	0.8	25.0	0.4–1.1	15	1.2	66.7	0.4–3.8
T40	16	115.7^M^	29.2	35.9–175.4	7	232.0^H^	71.7	118.2–550.8	15	218.8	109.8	27.3–946.5
T42	16	98.8^A,M^	24.7	59.7–155.5	7	155.0^H^	48.3	103.9–293.4	15	186.3^H^	122.0	74.9–991.0
T-*β*APB	16	214.5^M^	22.1	121.8–307.2	7	387.0^H^	60.1	222.8–778	15	405.1	113.1	116.6–1937

All values are expressed in pg/mL. H, M, and A mean significant (*P* < 0.05) with regard to HC, MCI and AD respectively. ^∗^means *P* < 0.01.

**Table 3:** Correlation between variables.

	UP A*β*1-40	DP A*β*1-40	CB A*β*1-40	UP A*β*1-42	DP A*β*1-42	CB A*β*1-42	MMSE	Right MTA
DP A*β*1-40	0.935^∗∗∗^	—	—	—	—	—	—	—
CB A*β*1-40	0.685^∗∗∗^	0.776^∗∗∗^	—	—	—	—	—	—
UP A*β*1-42	0.583^∗∗∗^	0.556^∗∗∗^	0.510^∗∗^	—	—	—	—	—
DP A*β*1-42	0.652^∗∗∗^	0.717^∗∗∗^	0.656^∗∗∗^	0.806^∗∗∗^	—	—	—	—
CB A*β*1-42	0.379^∗^	0.465^∗∗^	0.712^∗∗∗^	0.578^∗∗∗^	0.693^∗∗∗^	—	—	—
MMSE	−0.471^∗∗^	−0.395^∗^	−0.241	−0.485^∗∗^	−0.450^∗∗^	−0.274	—	—
Right MTA	0.321^∗^	0.280	0.257	0.530^∗∗^	0.510^∗∗^	0.353^∗^	−0.756^∗∗∗^	—
Left MTA	0.198	0.187	0.192	0.442^∗∗^	0.426^∗∗^	0.310	−0.686^∗∗∗^	0.894^∗∗∗^

Spearman coefficient for each pair of variables. ^∗∗∗^, ^∗∗^ and ^∗^mean *P* < 0.001, 0.01 and 0.05, respectively.

**Table 4:** Diagnostic features of the direct and calculated markers of the *β*APB.

Marker	Cutoff (pg/mL)		Sensitivity (>85%)	Specificity (>80%)	ROC (>0.80)
UP A*β*1-40	23.2 AD	AD/HC	40.0	93.8	0.59
	17.0 MCI	**MCI/HC**	**85.7**	68.8	**0.80 **
	22.5 AD	AD/MCI	40.0	71.4	0.35

DP A*β*1-40	63.8 AD	AD/HC	46.7	81.3	0.59
	63.8 MCI	**MCI/HC**	**85.7**	**81.3**	**0.81 **
	72.2 AD	AD/MCI	40.0	71.4	0.36

CB A*β*1-40	71.9 AD	AD/HC	53.3	87.5	0.65
	71.1 MCI	**MCI/HC**	**85.7**	**81.3**	**0.85 **
	211.3 AD	AD/MCI	13.3	100.0	0.40

UP A*β*1-42	10.28 AD	AD/HC	46.7	87.5	0.65
	9.2 MCI	**MCI/HC**	**85.7**	**81.3**	**0.90 **
	67.4 AD	AD/MCI	6.7	100.0	0.26

DP A*β*1-42	47.4 AD	AD/HC	46.7	87.5	0.62
	50.3 MCI	**MCI/HC**	57.1	**93.8**	0.79
	151.7 AD	AD/MCI	6.7	100.0	0.35

CB A*β*1-42	76.9 AD	AD/HC	40.0	100.0	0.74
	59.8 MCI	MCI/HC	100.0	50.0	0.72
	71.3 AD	AD/MCI	53.3	71.4	0.48

T40 (DP + CB)	132.7 AD	AD/HC	53.3	81.3	0.60
	132.7 MCI	**MCI/HC**	**85.7**	**81.3**	**0.83 **
	550.8 AD	AD/MCI	13.3	100	0.37

T42 (DP + CB)	115.8 AD	AD/HC	53.3	87.5	0.71
	103.3 MCI	MCI/HC	100	50	0.77
	113.7 AD	AD/MCI	53.3	57.1	0.41

T-*β*APB (T40 + T42)	235.5 AD	AD/HC	53.3	81.3	0.65
	235.5 MCI	**MCI/HC**	**85.7**	**81.3**	**0.84 **
	778.1 AD	AD/MCI	13.3	100	0.38

Highlighted in bold are the results that met the criterion considered suitable as figure in the heading. PPV: positive predictive value. NPV: negative predictive value. ROC: area under the receiver operating characteristic curve.
